# Textured Lead‐Free Ceramic with High Thermal Stability and Electrical Quality Factor

**DOI:** 10.1002/smll.202505193

**Published:** 2025-09-24

**Authors:** Aman Nanda, Sumanta Kumar Karan, Shankar Kunwar, Yongke Yan, Mark Fanton, Shashank Priya, Michael Lanagan, Bed Poudel

**Affiliations:** ^1^ Department of Materials Science and Engineering The Pennsylvania State University University Park PA 16801 USA; ^2^ Electronic Materials Research Laboratory Key Laboratory of the Ministry of Education and International Center for Dielectric Research School of Electronic Science and Engineering Xi'an Jiaotong University Xi'an 710049 P. R. China; ^3^ Department of Chemical Engineering and Materials Science University of Minnesota Minneapolis MN 55455 USA

**Keywords:** dielectric loss, energy harvesting, fatigue, lead‐free piezoelectric, textured ceramics, thermal stability, TSDC

## Abstract

A major challenge with (K,Na)NbO_3_ compositions is maintaining high piezoelectric figures of merit (FOM) in a wide range of temperatures and electric fields. A textured composition is demonstrated, K_0.48_Bi_0.02_Na_0.5_Nb_0.92_Sb_0.04_Zr_0.04_O_3_ incorporating 3 wt.% NaNbO_3_ templates, that exhibits a *d*_33_ of 570 pC/N and *k*_31_ of 0.4 at room temperature. Additionally, a high texture degree of ≈98% is achieved with the desired microstructural orientation. Electrical quality factor (*Q_e_*  =  1/tan δ) is effectively improved from 22 to 33 with 0.1 mol% MnO_2_ doping. The strain response (*S_max_/E_max_*) is calculated to be 450 pm V^−1^ under 40 kV cm^−1^, and *P*‐*E* and *S*‐*E* loops exhibited excellent fatigue resistance up to 10^6^ cycles. To validate the practical relevance, the energy harvesting performance is explored under both on‐resonance and off‐resonance conditions using vibration velocity measurement and a cantilever type energy harvester, respectively. The maximum output power recorded is ≈50 µW with a volume power density of ≈2 µW mm^−3^. The vibration velocity reached 0.26 m s^−1^ under a drive voltage of 30 V mm^−1^ at resonance. Through the combination of defect engineering (Mn doping) and microstructural engineering (texturing), the results confirm an excellent combination of electromechanical properties along with thermal stability and fatigue resistance in this material system.

## Graphical abstract

Textured (K,Na) NbO_3_‐based composition doped with MnO_2_ resulting in low dielectric loss, enhanced thermal stability, and fatigue resistance. The composition exhibits excellent energy harvesting performance under both on‐resonance and off‐resonance conditions. Defect and microstructural engineering Synergistically improves electromechanical properties, offering a promising lead‐free alternative for high performance piezoelectric application.

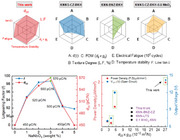

## 1 Introduction

Piezoelectric materials are designed with optimized combinations of electromechanical properties, thermal stability, and electrical loss depending upon the applications in transducers, sensors, energy harvesters etc.^[^ ^1^ ^]^ In off‐resonance application, high longitudinal piezoelectric charge constant (*d*_33_) and voltage constant (*g_33_*) are essential for energy harvesting and sensing.^[^ ^2^ ^]^ In contrast, in an on‐resonance application, the desired factors include high electromechanical coupling coefficient (*k*) and mechanical quality factor (*Q*_m_), low dielectric loss (*tanδ*), and minimal thermal sensitivity when driven under high alternating field.^[^ ^3^, ^4^ ^]^ The combination of desired properties is often achieved in the compositions near the morphotropic phase boundary (MPB) owing to good thermal stability and ultrahigh electromechanical response.^[^ ^5^ ^]^

The lead‐free (K,Na)NbO_3_ (KNN) piezoelectric composition exhibits polymorphic phase transformations (PPT) and low density (4.4 – 4.5 g cm^−3^).^[^ ^6^ ^]^ Inherently, KNN maintains a broad gap between intermediate ferroelectric‐ferroelectric phase transition temperature (≈200 °C) and very high Curie temperature (*T*_c_ ≈400 °C).^[^ ^5^, ^7^ ^]^ In conventional PPT systems, such as (K_0.5_Na_0.5_)NbO_3_ or (Bi_0.5_Na_0.5_)ZrO_3_, maintaining a balance between the magnitude of the piezoelectric constants (*d_33_* and *g_33_*) and their stability with respect to temperature is challenging over a wide temperature range. Initial attempts were made to enhance the piezoelectric constant by chemically modifying KNN system to converge the ferroelectric‐ferroelectric phase boundaries (T_OT_ and T_RO_) near room temperature as well as constructing diffused multiphase compositions.^[^ ^8^, ^9^ ^]^ These studies leverage the synergistic effect of multiple ferroelectric domain orientations and a broad phase transition peak to maintain the high magnitude of *d*_33_ and *d* ^*^_33_ (field‐dependent piezoelectric constant) over a wide temperature range.^[^ ^9^, ^10^ ^]^ Furthermore, textured microstructures with high degree of crystallographic orientation enhance the longitudinal piezoelectric constant while preserving the intrinsic properties.^[^ ^11^, ^12^ ^]^ This effect has been utilized in tuning the mechanical properties of Mg‐alloy heterostructure.^[^ ^13^ ^]^

The on‐resonance Figure of Merit (FOM) include factors *k*, *Q_m_*, and *tan (δ)* which can be enhanced by appropriate acceptor doping that pin domain walls and induce a “hardening” effect.^[^ ^14^ ^]^ The stability of vibration velocity in piezoelectric material when driven at resonance frequency depends upon the magnitude of applied field and the energy dissipated in the form of heat due to dielectric loss. Under high drive condition, the release of domain walls leads to softening of properties resulting in decline of resonant frequency as well as generation of heat.^[^ ^4^ ^]^ High dielectric loss further contributes to the heat generation leading to rapid overall degradation of properties. To maintain the on‐resonance FOM, it is necessary to control the dielectric loss in combination with lower thermal sensitivity of piezoelectric coefficient (*k_31_* vs *T*). Defect engineering through acceptor doping restricts the migration of oxygen vacancy under high drive field which helps in lowering dielectric loss and improving fatigue behavior.^[^ ^15^ ^]^

**Figure 1:**
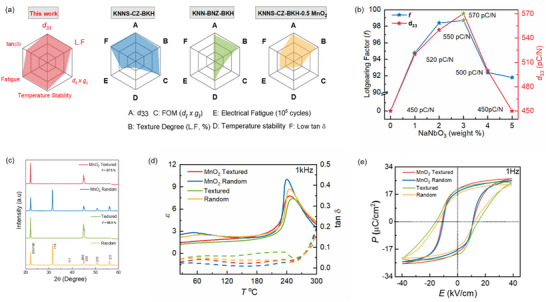
a) Comparison of properties of different textured KNN compositions reported with high *d_33_* (no report of tan(δ) on KNN‐BNZ‐BKH textured composition). b) Optimization of NN templates as a function of maximum texture degree (*f*) and piezoelectric constant (*d_33_*). c) XRD measurements showing the effect of 0.1 mol% MnO_2_ on the composition (KNN‐BSZ) as well as texture degree when textured with 3 wt.% NN templates. d) Temperature dependent measurement of relative permittivity (*ε_r_* versus *T*) and its respective dielectric loss at 1 kHz e) P‐E loop measurement for random, textured, and after modification with 0.1 mol% MnO_2._

A comparison of textured KNN compositions is provided in **Figure** **1**a. Typically, complex KNN compositions such as KNN‐CZ‐BKZ textured with NaNbO_3_(NN) templates exhibit high texture degree (94%) along with *d*_33_ (≈550–590 pC N^−1^).^[^ ^16^, ^17^ ^]^ However, they lack stability in terms of electric field (fatigue) and temperature which are more phase and defect chemistry dependent. Modification with acceptor dopants, such as 0.5 mol % MnO_2_, is known to resolve the issue of leakage current, but the impact on thermal stability remains unanswered.^[^ ^15^ ^]^

In this work, we report a modified KNN composition ceramic with a textured microstructure that exhibits an excellent combination of a high piezoelectric constant, temperature and electric field stability, and low dielectric loss, making it suitable for both on and off resonance piezoelectric energy harvesting applications. The composition given as: K_0.48_Bi_0.02_Na_0.5_Nb_0.92_Sb_0.04_Zr_0.04_O_3_ (KNN‐BSZ), exhibits high piezoelectric constant with enhanced thermal stability.^[^ ^18^ ^]^ Texture degree of KNN‐BSZ was optimized by template grain growth (TGG) process with an optimal amount of NN templates which results in improved piezoelectric and ferroelectric response. The effect of MnO_2_ on density, electrical properties, and texture degree was quantified, and further its influence on thermal stability and electric fatigue was investigated. A doping level of 0.1 mol% MnO_2_ reduced dielectric loss and produced a homogeneous microstructure with uniform grain size and higher density. The MnO_2_ modified textured composition was used in fabricating the cantilever‐type energy harvester operating at off‐resonance frequency. Vibration velocity measurements on rectangular samples were performed under resonance drive to evaluate the energy harvesting efficiency of the composition.

## 2 Results and Discussion

### 2.1 Piezoelectric and Dielectric Properties of <001> Textured Ceramics

The grain size, its distribution and texture degree in a fully sintered ceramic depends on the concentration of templates (Figure S3).^[^ ^11^, ^17^, ^19^ ^]^ Figure 1b illustrates the optimization of template amount as a function of texture degree (*f*) and small signal piezoelectric constant (*d*_33_). In this case, 2–3 wt.% NN templates help to achieve optimal texture degree (∼ 98%) along <001> direction resulting in increment of remanent polarization (*P_r_*) and *d*_33_.

With an addition of 3 wt.% NN templates, *d_33_* was improved from 450 to 570 pC N^−1^ and *P_r_* was enhanced from 16.6 to 19.7 µC cm^−2^ as compared to the unseeded random counterpart. Deviation from 3 wt.% ratio, degrades both piezoelectric and ferroelectric properties in terms of insufficient texture degree due to inadequate gap between template particles resulting in insufficient grain growth. Final grain size in a fully sintered ceramics is equal to the spacing between the template particles (*x_T_*) which depends on number frequency of templates (*f_T_*) formulated in Equation (1).^[^ ^11^ ^]^

(1) $$x_{T}={\left(6/\left(\pi f_{T}\right)\right)}^{1/3}$$

The above equation was derived by assuming uniform sized template particles placed equidistantly in 2D plane (matrix). *f_T_* strongly depends upon the volume of template loading (wt.% of NN seed) which is inversely proportional to final grain size.

Following the addition of 0.1 mol % MnO_2_, the texture quality remained unchanged, leading to an increase in relative density and improved dielectric loss (see Figure 1c and **Table** **1**). Figure 1d depicts the temperature‐dependent relative permittivity with its corresponding dielectric loss for both random and textured compositions. Textured ceramic after MnO_2_ modification manifested a *d*_33_ up to 590 pC N^−1^ with two‐fold decrement in dielectric loss at room temperature. Low dielectric losses were observed in the P‐E loop as a shrinkage in loop area in Figure 1e. With 0.1 mol% MnO_2_ addition, the area enclosed within P‐E loop reduced from 1.556 × 10^3^ to 1.086 × 10^3^ J m^−3^ in textured composition while in random composition the shrinkage was from 1.2 × 10^3^ to 936 J m^−3^. The reduction in the hysteresis of P‐E loops after MnO_2_ addition can be correlated with the reduced conductivity.^[^ ^20^, ^21^ ^]^ This reduction in conductivity can be attributed to annihilation of excess electrons originating from oxygen vacancies which were compensated by the defect complexes formed by Mn substitution on Nb site.^[^ ^22^, ^23^ ^]^ In addition, an asymmetric coercive field (($E_{c}^{+}-E_{c}^{-}$) ≈1.5 kV cm^−1^) was also observed in the MnO_2_ modified ceramics due to internal bias caused by the formation of defect dipoles.^[^ ^24^ ^]^ Interestingly, with texturing, the *ε_r_* of the composition exhibited converse behavior as compared to *d_33_* which is an added advantage for improving voltage constant (*g_ij_* = *d_ij_/ε_ij_*). This results in an exceptionally high off‐resonance FOM (*d_ij_* x *g_ij_*) making this material promising for energy harvesting.

**Table 1:** Summary of properties of random and textured composition before and after 0.1 MnO_2_ addition.

Composition	ρ [%]	*d_33_* [pC N^−1^]	*ε_r_*	*tan*[*δ*]	*P_r_* [µC cm^−2^]
Random	97	450	2150	0.043	16.6
0.1MnO_2_ Random	99	470	3000	0.024	18
Textured	97	570	1230	0.045	19.7
0.1MnO_2_ Textured	99	590	1400	0.030	19.7

**Figure 2:**
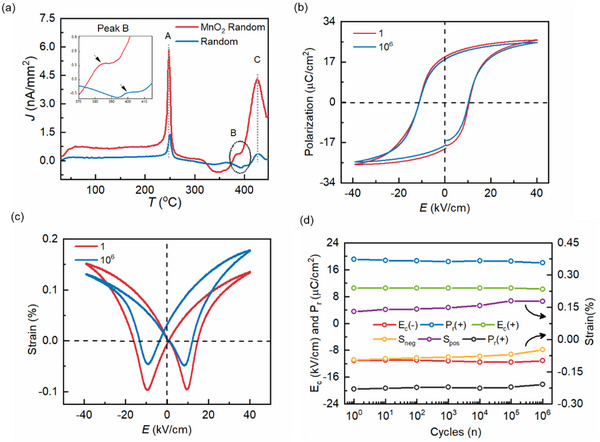
a) TSDC plot for 0.1 MnO_2_ modified and unmodified random ceramic. b) P‐E loop and c) bipolar strain loop measured for 10^6^ continuous cycles and its comparison with cycle −1. d) Magnitude of coercive field, strain, and polarization plotted against number of cycles per decade.

Since MnO_2_ is reported to be a potential hardener in KNN‐based compositions and its influence is evident in Figure 1e in the form of asymmetric coercivity and low loss, a more detailed mechanism can be examined using thermally stimulated depolarization current (TSDC) analysis.^[^ ^25^ ^]^ Literature suggests that TSDC spectra can be deconvoluted into peak “A”, “B”, and “C”, where “A” corresponds to intrinsic characteristic peak originating at phase transition temperature while “B” and “C” correspond to intragrain and intergrain migration of point defects, respectively (oxygen and alkali vacancy).^[^ ^26^ ^]^ **Figure** **2**a shows the depolarization current as a function of increasing temperature. Peak “A” for both 0.1 MnO_2_ modified and unmodified random composition aligns with the Curie temperature observed at ≈240 °C in Figure 1d. However, the intensity of J_max_ for MnO_2_ modified composition is notably higher as compared to unmodified KNN‐BSZ. The phenomena can be more likely due to the dissociation of defect dipole formed by Mn^4+^ substituting on Nb^5+^ site with oxygen vacancy ($Mn_{N_{b}}^{\prime }-V_{0}^{.}$ or $Mn_{N_{b}}^{\prime }-V_{0}^{..}\;-Mn_{N_{b}}^{\prime }$).^[^ ^27^ ^]^ Since defect dipole dynamics is a temperature‐driven process, its dissociation at higher temperature leads to generation of individual ions $Mn_{N_{b}}^{\prime }and\;V_{0}^{.}$ which increases charge carrier concentration. Dissociative ionization or defect ionization ultimately leads to enhanced conductivity at higher temperatures which can also be observed in peak “B” and “C”. Depolarization current (J_depol_) in both the composition can be formulated as:

(2) $$J_{depol}\left(Random\right)=J_{relaxation}+J_{space\;charge}$$

(3) $$\begin{array}{ccc} J_{\mathrm{depol}}\;\left(0.1\mathrm{Mn}O_{2}\;\mathrm{Rand}\mathrm{om}\right) & = & J_{\mathrm{rela}\mathrm{xati}\mathrm{on}}\;+J_{\mathrm{space}\;\mathrm{char}\mathrm{ge}}+J_{{\mathrm{Mn}}_{\mathrm{Nb}}^{I}}\\ & & +\;J_{\;V_{0}^{.}}+\;J_{\;V_{0}^{..}} \end{array}$$

Subsequently, the effect of defect dipole was also observed in the high voltage *P*‐*E* and *S*‐*E* loop measurements where cyclic loading and unloading of low frequency electric field drives the generation of electrostrain and polarization loops whose characteristics are influenced by domain wall dynamics. Since the defect dipole results in an internal bias at room temperature, it restricts the domain dynamics limiting the unidirectional compression/expansion of the material depending on field polarity.^[^ ^28^ ^]^ This also leads to a higher magnitude of coercive field for switching the domains.

Prior studies suggest that oxygen vacancy ($V_{0}^{.}$ and $V_{0}^{..}$) cluster at domain walls restricting the switching of domains under external electric field resulting in serious decline in magnitude of *P_r_* and *S_max_* in *P*‐*E* and *S*‐*E* loops.^[^ ^29^, ^30^ ^]^ Figure 2b,c shows the comparison of *P*‐*E* and *S*‐*E* loops measured with 1 Hz frequency drive field for 1 and 10^6^ cycles for 0.1 MnO_2_ textured ceramic. *P*‐*E* loop in Figure 2b shows fatigue‐free behavior and the deviation in *P_r_* and *E_c_* is negligible after 10^6^ cycles. Conversely, in *S‐E* loop, *S_pos_* maintains its magnitude while *S_neg_* shows a fatigued behavior likely due to restriction of non‐180° domain mobility due to continuous cyclic loading of electric field.^[^ ^29^ ^]^ The formation of defect dipole controls the agglomeration of oxygen vacancy resulting in unrestricted motion of domain walls. In the case of 0.1 MnO_2_ modified random ceramic, the presence of R‐O‐T coexistence coupled with Mn^4+^ doping, neutralizes the migration of oxygen vacancies in form of defect complexes like (${\mathrm{Mn}}_{\mathrm{Nb}}^{I}$‐ $V_{0}^{.}$) and (${\mathrm{Mn}}_{\mathrm{Nb}}^{I}$‐ $V_{0}^{..}-\;{\mathrm{Mn}}_{\mathrm{Nb}}^{I}$) which provides multiple advantages like low dielectric loss and fatigue‐free behavior.^[^ ^15^ ^]^

### 2.2 Phase and Microstructure of Textured Ceramics

Figure S1 (Supporting Information) illustrates Rietveld refinement of X‐ray diffraction data for the quantitative estimation of crystallographic phase content, alongside the temperature‐dependent relative permittivity measurement (*ε_r_* v/s *T*) for both random compositions. KNN‐BSZ contains phases R3m, Amm2, and P4mm in volume fraction of 6%, 73%, and 21% respectively while the distribution in 0.1 MnO_2_ KNN‐BSZ is 10%, 79%, and 11% respectively (summarized in Table S1, supporting information). The plot of *ε_r_* versus *T* displays a single broad peak corresponding to ferroelectric‐ferroelectric phase transition near room temperature and the Curie temperature ≈240 °C. This indicates a diffused and multiphase coexistence which contributes toward high piezoelectric and dielectric properties at room temperature along with reduced thermal sensitivity of physical properties.^[^ ^31^ ^]^ With the addition of 0.1 mol% MnO_2_, the quantitative phase fraction along with Curie temperature remains unaffected and dielectric loss is reduced. In addition, the plot of *ε_r_* versus *T* manifests no thermal hysteresis between the heating and cooling cycle of relative permittivity measurement which also confirms first‐order displacive phase transition in ferroelectric‐paraelectric phase.^[^ ^32^, ^33^ ^]^

**Figure 3:**
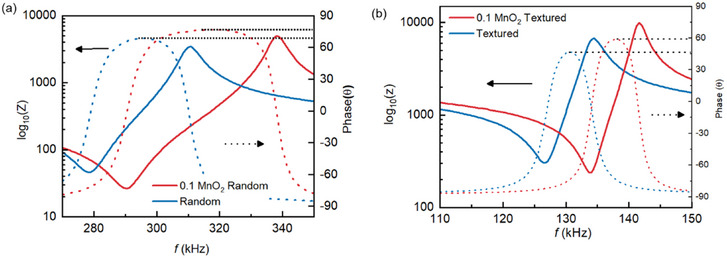
Impedance and phase as a function of frequency providing resonance and antiresonance frequency that is used for *k_31_* and *d_31_* calculation, a) random composition and b) textured ceramic.

**Figure 4:**
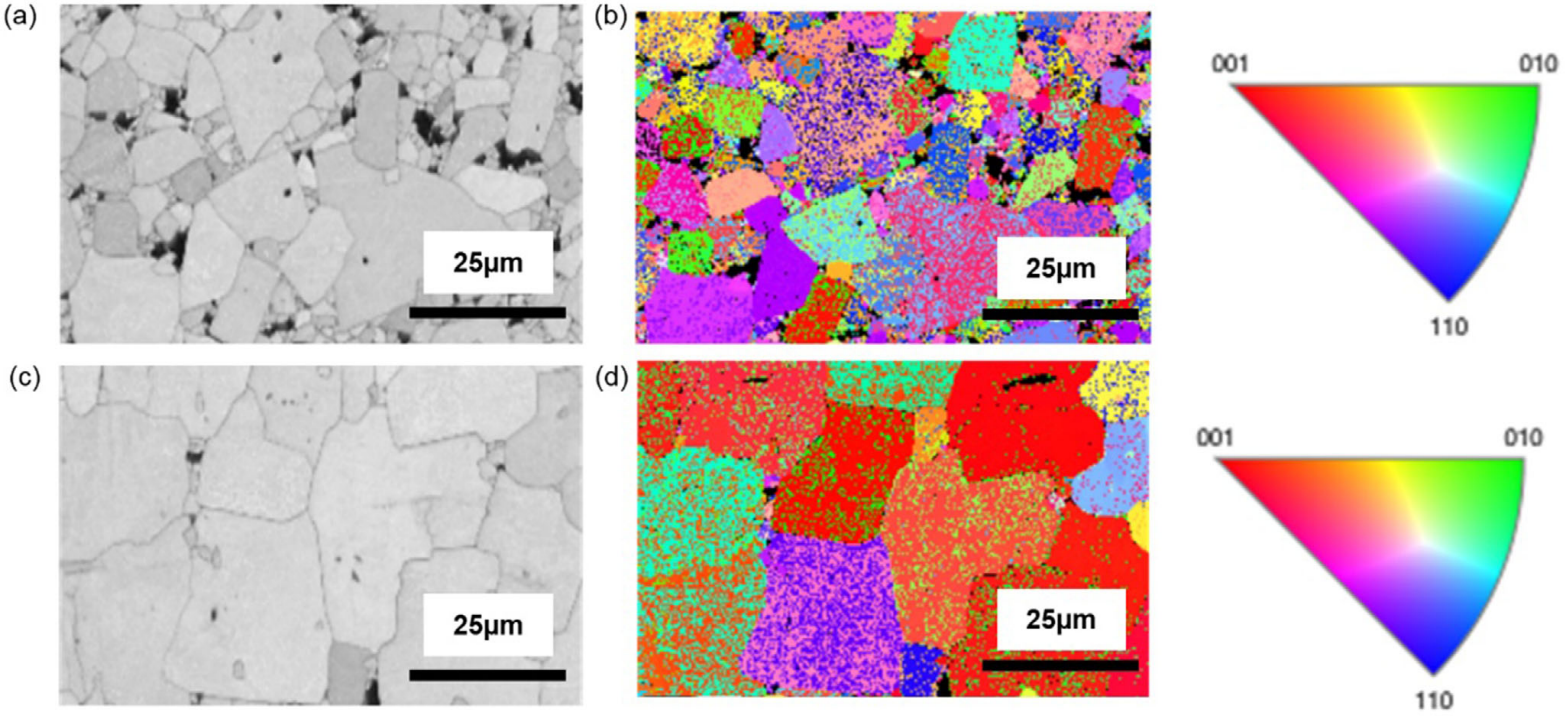
a) Band contrast image of random ceramic with definitive grain boundary and b) its EBSD measurement along z direction with scale. c) band contrast image of textured ceramic and d) its EBSD measurement with scale.

Further, to understand the extrinsic contribution to piezoelectricity and texture mechanism, microstructural characterization on the fractured surface was conducted as illustrated in Figure 4. Addition of MnO_2_ leads to more uniform grain size distribution and less porosity as shown in Figure S2 (Supporting Information), resulting in high relative density (≈99%). Effect of liquid phase on promoting sintering has been studied in multiple material system^[^ ^34^ ^]^ MnO_2_ has been reported as a potential sintering aid that facilitates the formation of denser microstructure through liquid phase.^[^ ^35^, ^36^ ^]^ Denser microstructure leads to enhanced bulk impedance and lower phase mismatch resulting in higher relative permittivity.^[^ ^37^ ^]^ Impedance spectra shown in **Figure** **3**a,b confirms that in presence of MnO_2_, the bandwidth (*f_a_*‐*f_r_*) is considerably high which results in higher *k_p_* and *k_31_* in random and textured compositions respectively. The improved bandwidth is due to increase in the electromechanical coupling of the composition due to better poling (reduced conduction due to pinning of domains) and enhanced piezoelectric coefficient due to improved densification.^[^ ^38^ ^]^ Summary of the properties are listed in Table 1. Further an increment in the phase angle (θ) of the impedance is also observed likely due to reduction in resistive leakage originating from poor microstructure and porosity.

Textured ceramics after templated grain growth possess larger grain size as compared to random counterpart. **Figure** **4**a,b presents phase contrast and EBSD spectra respectively, measured along “*c*” direction for random KNN‐BSZ. Figure 4c,d presents a similar measurement for textured grain. However, the size of textured grains are larger and unidirectionally oriented along <001> direction as confirmed by EBSD. Grains are well aligned in casting direction after high temperature multistep sintering process as confirmed in Figure S2 (Supporting Information). Similar conclusions can be drawn from Figure S5 (Supporting Information) for MnO_2_ modified compositions. The grains maintained their orientation after modification with MnO_2_ and processed using multistep sintering. The process of grain growth on NN templates (TGG) for multi‐element modified KNN matrix is reported to take place in three different stages: reaction between the template and matrix interface and formation of liquid phase, rapid growth of template particle by leveraging liquid phase and finally the restriction of growth due to impinged surface.^[^ ^39^ ^]^ These stages are time‐dependent and the growth mechanism can be both homoepitaxy and heteroepitaxy followed by topotactic transition.^[^ ^40^ ^]^ In a similar fashion, the optimization of texture degree with appropriate amount of NN templates (3 wt.%) can be interpreted from the microstructural perspective. In the case of lower amount/frequency of templates (1 and 2 wt.%), there is significant spacing between the templates resulting in abnormal grain growth (AGG) in matrix during stage ‐III of TGG process which drives smaller random grains. Conversely, with higher frequency or closer spacing of templates (4 and 5 wt.% of templates), TGG is restricted by faster grain boundary impingement as mentioned in Figure S3 (Supporting Information). The final grain size is affected by the frequency of template which is controlled by the wt.% of NN seeds and aligns well with Equation (1). In all these cases, the surface of NN templates acts as the site of heterogeneous nucleation and it has to be physically and chemically stable during all the stages.^[^ ^40^, ^41^ ^]^ Reaction between the template and matrix at different temperatures are mentioned in Figure S3 (Supporting Information). The template remains stable and intact providing enough room for growth till 1100 °C which is the sintering temperature.

### 2.3 In Situ Thermal Stability Analysis of Textured Ceramic

**Figure 5:**
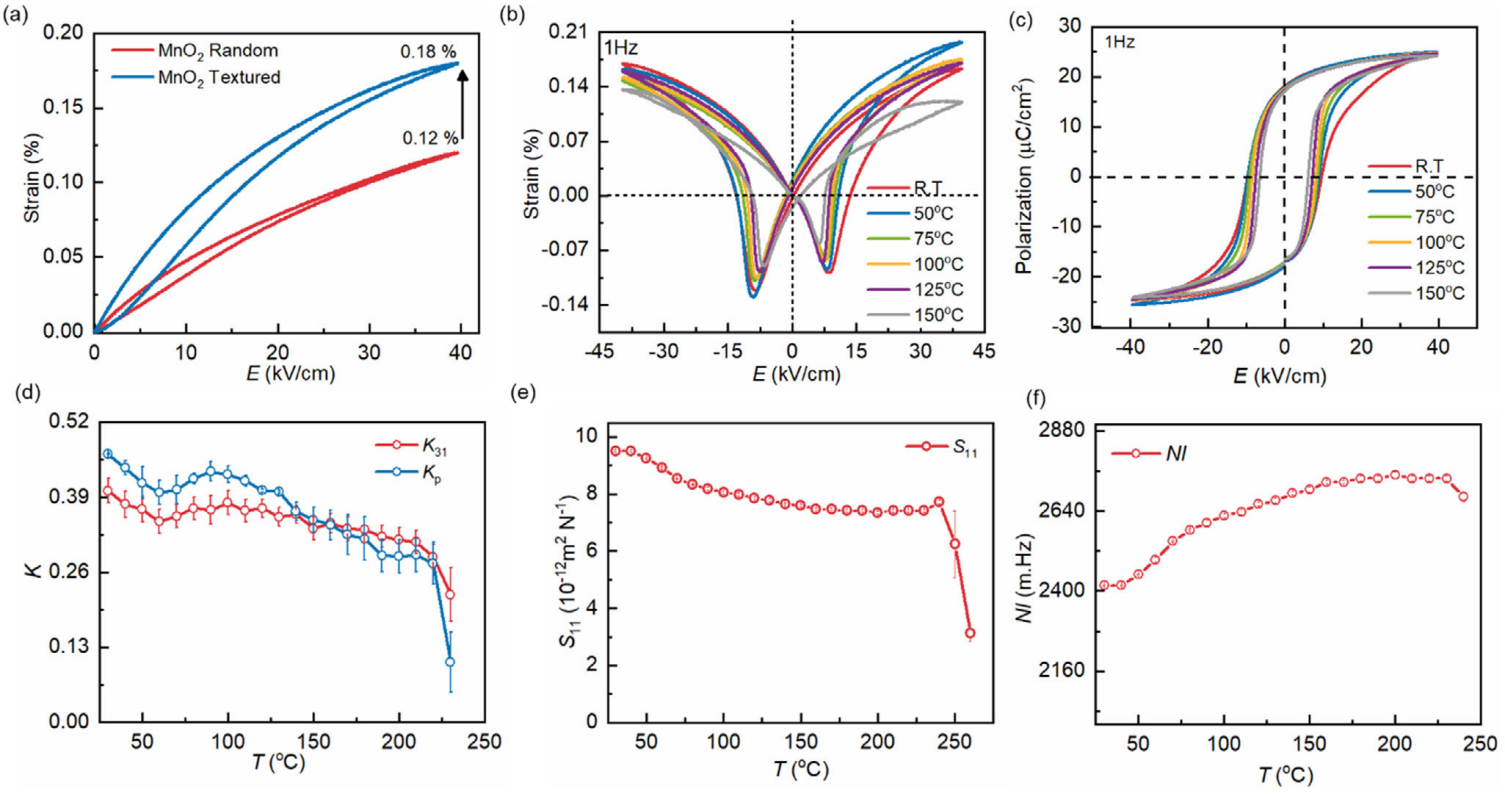
a) Comparison of unipolar strain loop between 0.1 mol% modified MnO_2_ random and textured ceramics, temperature‐dependent measurement of b) Strain and c) Polarization as a function of electric field, d) Stability of electromechanical coupling *k_31_* and *k_p_* with change in temperature. Variation of e) elastic compliance (S_11_) and f) frequency constant (*Nl*).

**Figure** **5**a depicts the comparison of unipolar strain between the random and textured 0.1 MnO_2_ KNN‐BSZ. Electrostrain improved from 0.12% to 0.18% after texturing. The magnitude of S_max_/E_max_ (d^*^_33_) calculated at 40 kV cm^−1^ was found out to be 300 and 450 pm V^−1^ for random and textured ceramics, respectively.

**Figure 6:**
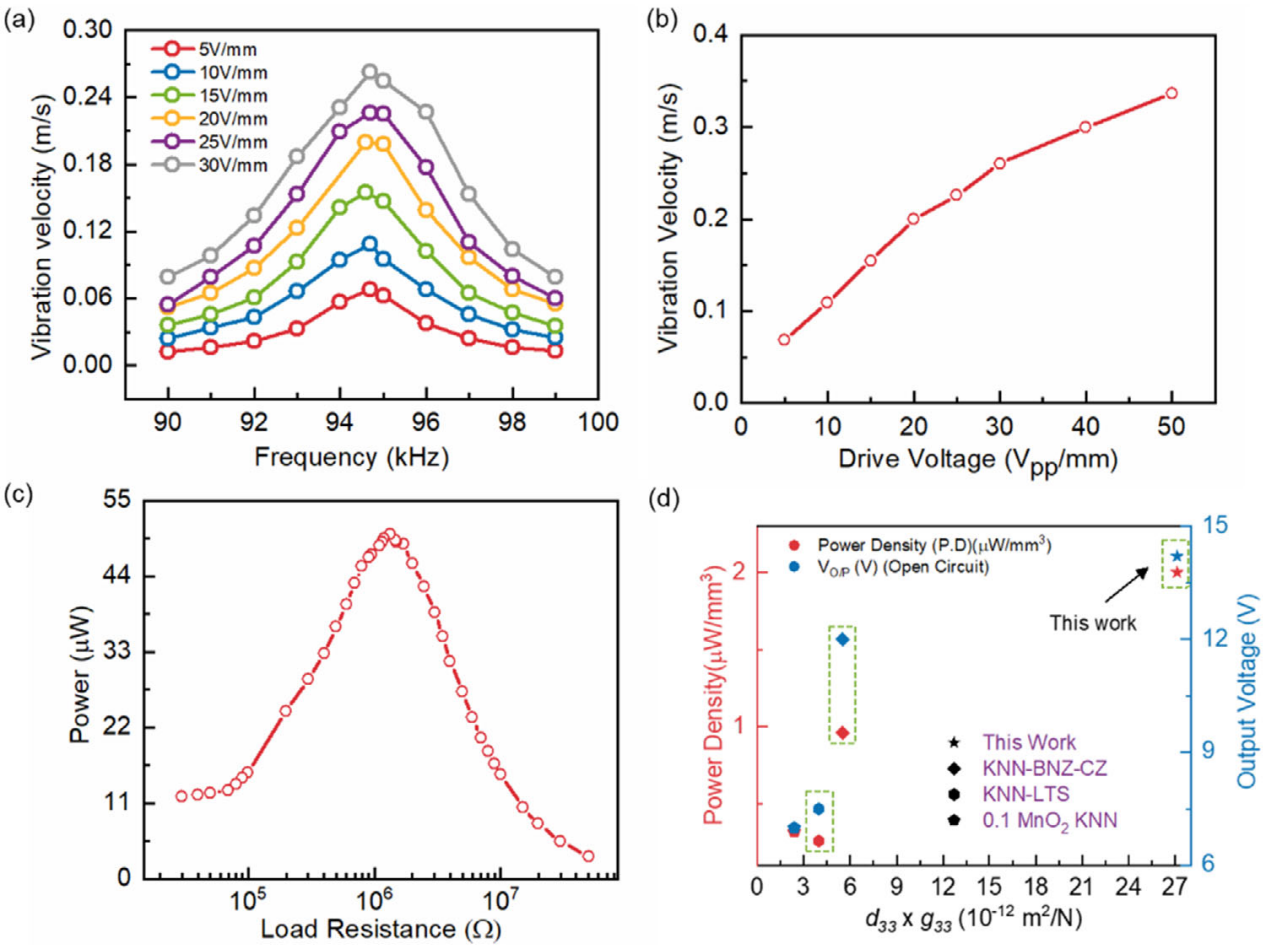
a) Resonant frequency determination for 0.1 MnO_2_ textured ceramic when excited at different drive voltages, b) Plot of vibration velocity at resonance for different drive voltages, c) Output power recorded using impedance matching method, and d) Summary of power density for different lead‐free compositions summarized as a function of their FOM.

In situ thermal stability of electrical properties is an important aspect to be considered for transducers and sensors applications. Piezoelectric properties like *d_31_* and electromechanical coefficient (*k_31_*) are higher near phase boundary. *k_31_* and *d_31_* are correlated as:^[^ ^42^ ^]^

(4) $$k_{31}^{2}=d_{31}^{2}/\;\left(s_{11}^{E}\varepsilon_{33}^{T}\right)$$

where $s_{11}^{E}$ is the elastic compliance under constant electric field and $\varepsilon_{33}^{T}$ is relative permittivity at stress‐free condition. Further, the sensitivity of coupling coefficient (*k_31_* and *k_p_*) and s*_11_* are shown in Figure 5d,e, respectively. The magnitudes of *k_31_* and *k_p_* pose a small dip which aligns with the trend of broad peak observed in *ε_r_ ‐T* measurement between (50 – 75) °C likely due to diffused phase transition (Figure S1, Supporting Information).^[^ ^29^ ^]^ In order to confirm the reliability of results, data from 8 different samples from multiple batches were averaged and represented with standard deviation (error bar) in **Figure** **6**d. All the samples exhibited the similar trend of *K_31_* as a function of temperature. The 95% confidence intervals including the upper (UCI) and lower (LCI) limit are mentioned in Table S2 (Supporting Information). The *s_11_* and length frequency constant (*Nl*) do not reflect any phase transition with a change in temperature rather all the properties fall after Curie temperature, as expected. The difference in the sensitivity of electrical properties is more likely due to the changes in the impedance spectrum of the ceramic as a function of temperature resulting in the variation of the position of *f_r_* and *f_a_* as well as bandwidth (*f_r_* – *f_a_*). The precision in measuring the *f_r_* and *f_a_* as a function of temperature is critical toward identifying the absolute value of electromechanical constants.

The diffused R‐O‐T phase structure leads to less sensitive electrical properties. Converse and direct piezoelectric properties have different thermal sensitivity depending upon stability of domains under bipolar electric field and phase transition, respectively.^[^ ^15^ ^]^ Figure 5b,c represents the bipolar polarization and strain loop measurement on 0.1 mol% MnO_2_ textured ceramic at different temperatures. The bipolar strain loop is stable till 125 °C and there is no significant change in remanent polarization as a function of temperature in P‐E loop. The coercive field (*E_c_*) reduces with temperature due to easier domain switching at high temperature.^[^ ^43^ ^]^

### 2.4 Energy Harvesting Performance of Textured Ceramic (On and Off Resonance Frequency)

Demonstration of on‐resonance and off‐resonance energy harvesting properties of 0.1MnO_2_ KNN‐BSZ textured composition are presented in Figure 6. The FOM for energy harvesting at on‐resonance and off‐resonance can be estimated as:^[^ ^1^ ^]^

(5)

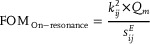



(6)

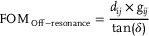




The magnitude of electromechanical constants are summarized in **Table** **2**. To evaluate the on‐resonance energy harvesting properties, piezoelectric vibration characteristics (*v*) were measured along the fundamental length direction of sample poled along thickness direction. The vibration velocity was measured up to 0.26 m s^−1^ at resonance frequency (94.7 kHz) with 30 V mm^−1^ drive voltage. Theoretically *v* can be calculated as:^[^ ^44^ ^]^

(7) $$v=\frac{4}{\pi }\times \frac{d_{ij}\times Q_{m}\times E}{\sqrt{S_{ij}^{E}\times \rho }}$$

**Table 2:** Comprehensive properties of material constant of textured compositions.

Material	*E_c_* [kV cm^−1^]	Dielectric		Coupling coefficient			Piezoelectric constant				Elastic Constant		Density	Refs.
		*tan* [*δ*]	*ε_r_*	*K_p_*	*K_31_*	*K_t_*	*d_33_ d_31_* (10^−12^ C/N)		*g_33_ g_31_* (10^−3^ Vm/N)		$s_{11}^{E}\;s_{11}^{D}$ (10^−12^ m^2^/N)		g/cc	
Textured Mn‐PMN‐PT	5‐6	0.005	2200	0.63	0.44	0.54	517	207	26.5	10.6	11.5	9.3	8	[50, 51]
Textured Mn‐PMN‐PZT (1)	9‐10	0.003	1595	0.83	0.56	0.55	600	239	35.4	16.9	15.6	11.6	7.8	[50, 52]
Textured Mn‐PMN‐PZT (2)	9‐10	0.003	2450	0.83	0.56	0.55	817	381	36.6	17.5	20.9	14.2	7.54	[50, 53]
Textured Mn‐KNN‐BSZ	10‐11	0.03	1400	0.52	0.42	0.41	590	160	46	12.5	12.5	10.5	4.5	This work

 which is proportional to FOM (d_*ij*_ × *Q_m_*) governing on‐resonance performance. Figure 6a presents measurement of vibration velocity with an increasing magnitude of applied bias from 5 to 30 V mm^−1^. The resonance observed at 94.4 kHz is not sensitive to applied voltage which is advantageous for numerous on resonance applications. Moreover, the low theoretical density (4.4 g cc^−1^) helps achieve comparable magnitude of vibration velocity with soft PZT irrespective of low d_33_ and Q_m_.^[^ ^6^ ^]^ The vibration velocity at resonance starts to saturate with the increase in drive field as can be seen in Figure 6b.

Off‐resonance piezoelectric energy harvesting (PEH) was performed with an elephant‐shaped beam structure tested at 1 g excitation with 13 g tip mass.^[^ ^45^ ^]^ The design of energy harvester is mentioned in Figure S7 (Supporting Information). The maximum output (O/P) power (peak to peak) measured via impedance matching method was found to be ≈50 µW with a ≈14V_pp_ O/P voltage in open circuit condition at 130 Hz (Figure 6c). The power density was calculated to be ≈2 µW mm^−3^ which is ≈2 times higher than the previously reported KNN system based on the literature values (Table S3, Supporting Information). The 0.1mol% modified MnO_2_ textured ceramic provides promising performance for both on‐resonance as well as off‐resonance electronics application along with enhanced thermal stability and lower dielectric loss.

A survey of state‐of‐the‐art output power density for different lead‐free (KNN) compositions are summarized in Figure 6d as a function of their respective FOM.^[^ ^46^, ^47^, ^48^ ^]^ The 0.1 mol% modified MnO_2_ textured ceramic yields a maximum electrical power density and output voltage as compared to other lead‐free counterparts due to high FOM. The off‐resonance FOM (*d_33_* x*g_33_*) is exceptionally high in textured composition, likely due to crystal anisotropy resulting in 1) enhancement of d_33_ and 2) decline in the magnitude of *ε_r_* which subsequently leads to very high voltage constant (g_33_). Such textured compositions where crystal anisotropy leads to high charge constant coupled with exceptionally high voltage constant can have wide range of future applications comparable with existing Pb‐based compositions. The comparison of real time implementation of cantilever‐type PEHs are summarized in Table S4 (Supporting Information). To draw more detailed insight, Table 2 summarizes a comprehensive collection of dielectric, piezoelectric and elastic properties which were used for finite element analysis of tonpilz transducers which also helped developing a material design criteria of ring stack devices.^[^ ^49^, ^50^ ^]^ Low density combined with good piezoelectric constants makes textured KNN composition a competent material for future Pb free transducer application.

## 3 Conclusion

Piezoelectric properties in KNN‐BSZ ceramics were improved by texturing with NN templates and a systematic study on texture development was conducted to understand the microstructural and phase evolution. Addition of 3 wt.% NN templates helped attain a high texturing degree ≈98% which improved d_33_ and P_r_ by 25%. Dielectric loss was successfully reduced by two orders of magnitude with 0.1 mol % MnO_2_ addition which also contributed to higher density. The 3 wt.% NN templates based textured KNN‐BSZ ceramic modified with 0.1 mol% MnO_2_ exhibited d_33_ of 580–600 pC N^−1^ at room temperature with excellent thermal stability for k_31_ and bipolar strain loops. Further, the P‐E and S‐E loops show fatigue free behavior after 10^6^ fatigue cycles which indicate a higher lifetime. To validate the energy harvesting performance of these materials at on‐resonance and off‐resonance, vibration velocity and output electrical power were measured on cantilever beam structure respectively. The vibration velocity was measured up to 0.26 m s^−1^ with 30 V mm^−1^ drive voltage at resonance while the maximum output electrical power was reached up to ≈50 µW with ≈2 µW mm^−3^ power density. Overall, the composition possesses an ideal combination of electrical properties along with reduced thermal sensitivity of FOM and good fatigue resilience. Finally, based on the different comparison tables, the demonstrated material has a strong potential to be a competing candidate for transducers, energy harvesters and sensors.

## 4 Experimental Section

### Material Synthesis and Characterization

(K_0.48_Na_0.5_Bi_0.02_)(Nb_0.92_Sb_0.04_Zr_0.04_)O_3_ (KNN‐BSZ) was prepared by conventional solid state synthesis route as shown in Figure S6a (Supporting Information). High purity K_2_CO_3_ (Sigma Alderich, 99%), Na_2_CO_3_ (Sigma Alderich, 99.5%), Nb_2_O_5_ (Alfa Aesar, puratronic 99.9985%), Sb_2_O_3_ (Alfa Aesar, 99.6%), Bi_2_O_3_ (Alfa Aesar, 99%), and ZrO_2_ (Alfa Aesar, 99.7%) powders were weighed in the stoichiometric ratio and ball milled in ethanol media to obtain homogeneous mixture. The mixture was dried and calcined at 850 °C and ball milled with 0.1 mol% MnO_2_ (Alfa Aesar, 99.9%). Ceramic tapes were prepared using tape casting with doctor blade technique at a speed of 4.5 cm s^−1^.^[^ ^17^ ^]^ The synthesis process of textured ceramics is schematically described in Figure S6b (Supporting Information). The textured ceramics were prepared through templated grain growth process with a controlled weight percentage (wt.%) of the templates mixed in the slurry used for tape casting. The templates used were NaNbO_3_ (NN) platelets obtained through molten salt synthesis followed by topochemical conversion.^[^ ^54^ ^]^ Both the random and textured ceramics were sintered with multistep sintering profile depicted in Figure S6c (Supporting Information). Initially, the sintering temperature was raised to 1200 °C at 5 °C min^−1^ and then quickly cooled down to 1090 °C at 10 °C min^−1^ with dwelling time of 10 h. Powder XRD was performed with a scan rate of 3 deg min^−1^ for Rietveld refinement (Melvin Panalytical Empyrean ‐III) and all the FESEM and EBSD were measured using backscattered detectors (Thermo Scientific Fischer, Oxford instruments). All the ceramic samples were prepared and measured according to IEEE standard.^[^ ^42^ ^]^ Textured degree was calculated through Lotgering factor which is evaluated as:^[^ ^55^ ^]^

(8) $$L.F\;\left(f\right)=\frac{P-P_{0}}{1-P_{0}}$$

where P is ratio of summation of peaks of preferred orientation to the summation of peaks corresponding to all directions in textured ceramics; P_0_ is the equivalent ratio for random ceramics. Volume fraction of each phase in the random ceramics was calculated by quantitative analysis of peak fitting through Rietveld refinement analysis in MAUD. The scan rate of XRD measurement for Rietveld refinement analysis was fixed at 3 degree min^−1^ to acquire detailed information of crystal structure. Microstructural characterization was conducted using Scanning Electron Microscope (SEM, ThrmoFisher Scientific, Apreo). The measurement of both FE‐SEM and EBSD were done through backscattered detectors to acquire information on grain distribution based on difference in contrast. Densities of the sintered ceramics were measured by Archimedes method and further relative density was calculated in reference to theoretical density of KNN.^[^ ^56^ ^]^

### Electrical Measurements

Impedance as a function of frequency was measured using Impedance Analyzer (Keysight E4990A) and the electromechanical coupling factor was calculated from the resonance (*f_r_*) – antiresonance (*f_a_*) modes. Temperature‐dependent relative permittivity (*ε_r_ – T*) was measured through LCR meter (Keysight E4980A) combined with a temperature‐controlled chamber. Thermal stability measurements were performed using the same setup synchronized with an impedance analyzer. The rate of increase of temperature was kept constant for both measurements at 5 °C min^−1^. Strain and fatigue measurements were carried out with the P‐E tracer combined with amplifier (Radiant Ferroelectrics). Thermally Stimulated Depolarization Current (TSDC) measurement was carried out on the samples poled at 150 °C under 2 kV mm^−1^ in Si oil bath for 10 min. Electrodes were shorted before measurements to get rid of hetero charges accumulating near the electrode. Temperature ramping was carried out at 5 °C min^−1^ and current was measured with a picoamp meter.

### Energy Harvesting and Vibration Velocity Measurement

Cantilever type energy harvester was designed with mode rectangular textured ceramic attached to the stainless‐steel elephant‐type transducer. The schematics of energy harvester are shown in Figure S7a (Supporting Information). Under an optimized tip mass, the harvester was induced with mechanical vibration through mechanical shaker and output voltage was recorded using an oscilloscope. The conventional sinusoidal waveform was generated through Siglent SDG 1025 function generator (F.G) and the signal was fed to the mechanical shaker. The O/P (output) voltage from the ceramic was monitored using Keysight DSOX4024A digital storage oscilloscope. The maximum O/P power was optimized through impedance matching method with a variable resistance in parallel. Vibration velocity measurement was carried out using Polytec laser doppler vibrometer. The schematics of vibration velocity measurement is illustrated in Figure S7b (Supporting Information). The sample was electrically driven at resonance (≈94.4 kHz) with F.G and amplifier (NF electronic Instruments 4025). The resulting mechanical vibrations were captured through the Doppler principle of the vibrometer. The maximum acquisition frequency of the tool is 100 kHz and the measurements were conducted using bandpass filter set to 50 kHz, enabling a working frequency of 50 – 100 kHz. The sample's resonant frequency fell within the range of acquisition limit ensuring reliable and conclusive measurement.

## Conflict of Interest

The authors declare no conflict of interest.

## Supporting information



Supporting Information

## Data Availability

Data sharing is not applicable to this article as no new data were created or analyzed in this study.
