# Supplementary material for: Textured Lead‐Free Ceramic with High Thermal Stability and Electrical Quality Factor
Source: Small. 2025 Sep 24;21(44):e05193. doi: 10.1002/smll.202505193 (PMC12590523; doi:10.1002/smll.202505193)
Supplement: Supplementary file 1 — Supporting Information [file SMLL-21-e05193-s001.docx]

**Textured Lead-Free Ceramic with High Thermal Stability and Electrical Quality Factor**

Aman Nanda^1*^, Sumanta Kumar Karan^1^, Shankar Kunwar^1^, Yongke Yan^2^, Mark Fanton^1^ Shashank Priya^3*^, Michael Lanagan^1*^ and Bed Poudel^1*^

^1^ Department of Materials Science and Engineering, The Pennsylvania State University, University Park, PA, 16801

^2^ Electronic Materials Research Laboratory, Key Laboratory of the Ministry of Education and International Center for Dielectric Research, School of Electronic Science and Engineering, Xi’an Jiaotong University, Xi’an 710049, P.R. China

^3^ Department of Chemical and Materials Engineering, University of Minnesota, Minneapolis, MN, 55455

**Supporting Information**

1. **Microstructure and Phase Analysis of Random Composition**


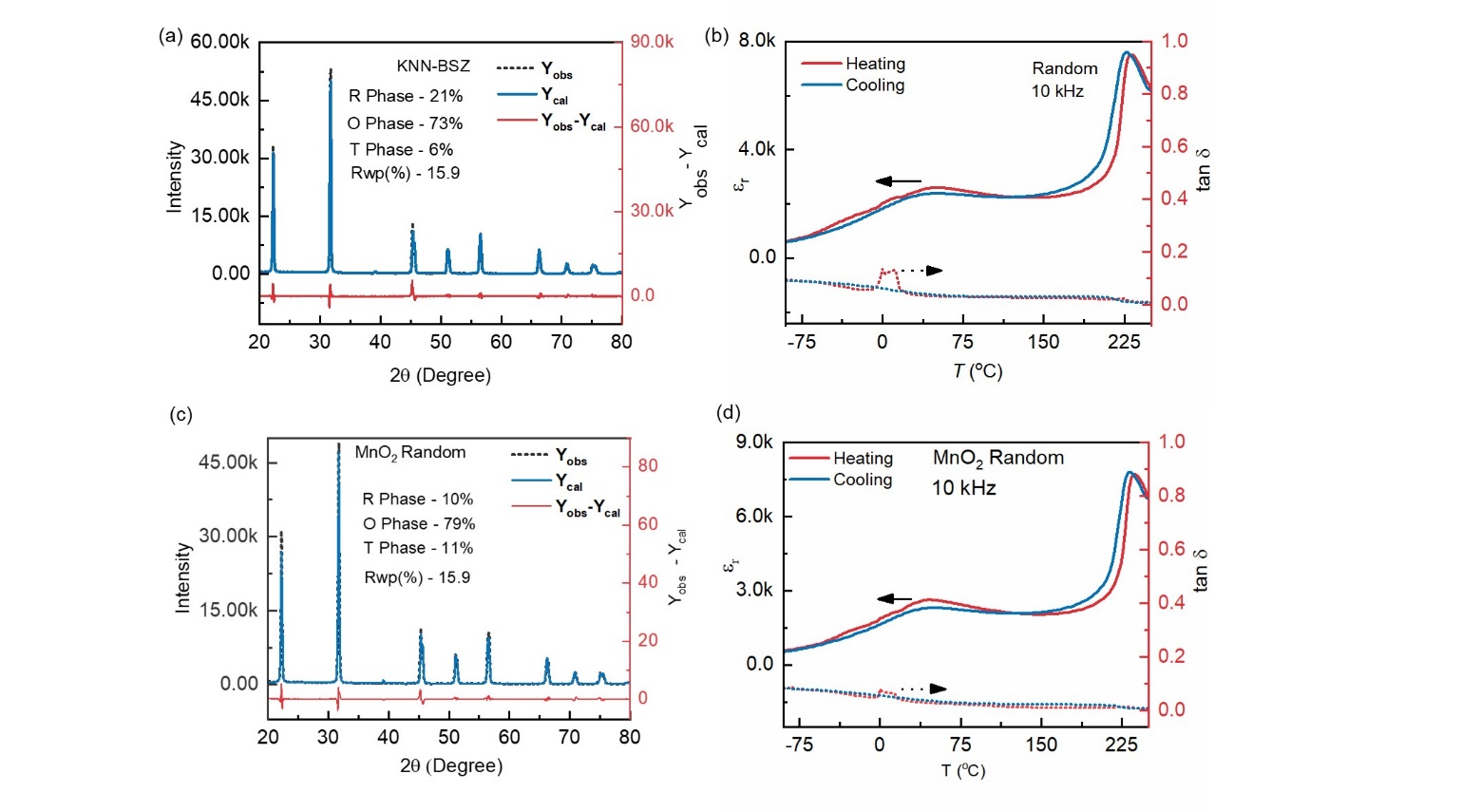


Figure S1. Retvield fitting of (a) KNN-BSZ and (c) 0.1MnO_2_ KNN-BSZ *ε_r_ v/s T* and its corresponding *tan(δ)* for heating and cooling cycle for (b) KNN-BSZ and (d) 0.1MnO_2_ KNN-BSZ

**Table S1. Summary of lattice parameters drawn from Retvield Refinement**

| Phase | Random | Fraction | 0.1 MnO_2_ | Fraction |
| --- | --- | --- | --- | --- |
| Tetragonal (T)  a = b < c  α = β = γ = 90^o^ | a = 3.9766  b = 3.9766  c = 4.0184 | 21% | a = 3.9768  b = 3.9768  c = 4.0145 | 10% |
| Orthorhombic (O)  a ≠ b ≠ c  α = β = γ = 90^o^ | a = 3.9801  b = 5.6598  c = 5.6648 | 73% | a = 3.9792  b = 5.6588  c = 5.6669 | 79% |
| Rhombohedral (R)  a = b = c  α = β = 90^o^ γ = 120^o^ | a = 5.6378  b = 5.6378  c = 6.9003 | 6% | a = 5.6291  b = 5.6291  c = 6.9190 | 11% |


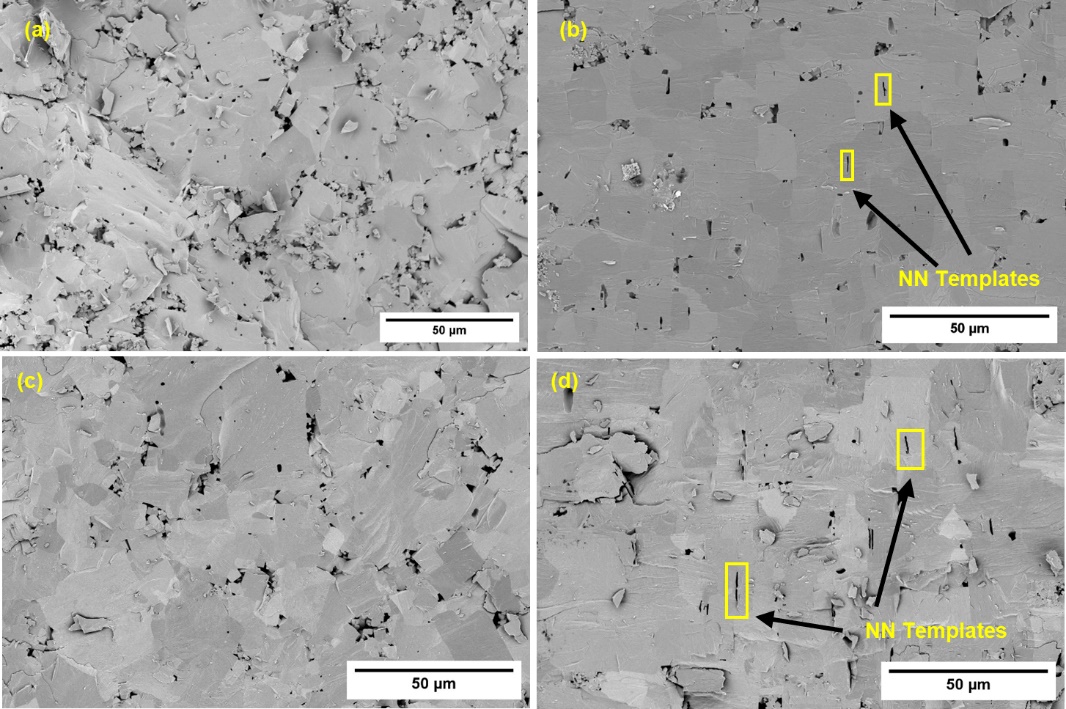


Figure S2. FE-SEM of (a) Random, (b) Textured, (c) 0.1 MnO_2_ random and (d) 0.1 MnO_2_ Textured

1. **Optimization of Template Amount and Microstructure**


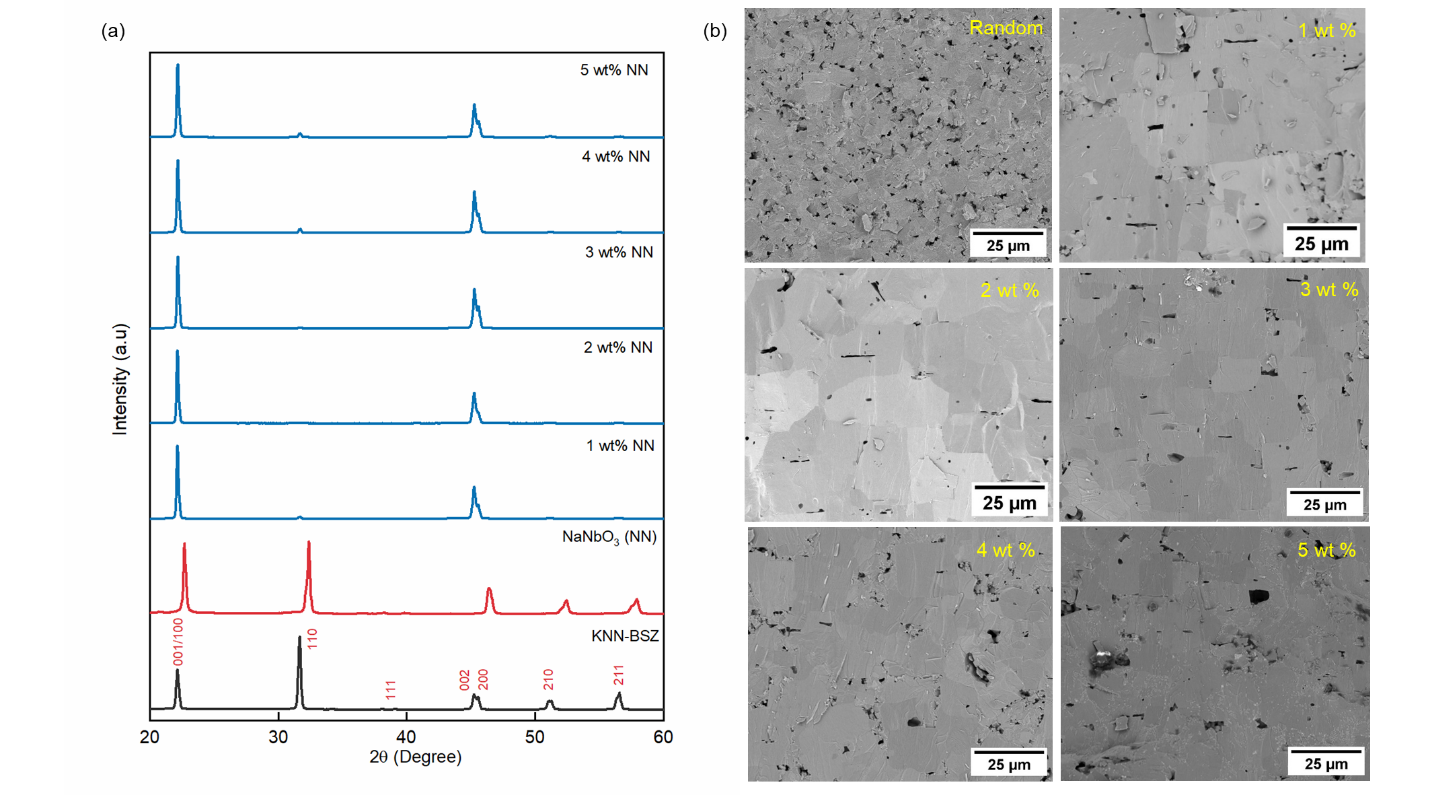


Figure S3. Optimization of texture degree. (a) XRD pattern demonstrating the degree of texture with seed addition. (b) SEM micrographs of KNN-BSZ with a range of NN seed addition.

To study the template – matrix interaction and the growth mechanism as a function of temperature FE-SEM was conducted on fractured cross section of thermally treated ceramics at 900^o^C, 1000^o^C, 1100^o^C and 1200^o^C for 15 min shown in figure S.I-3. The surface of the template grows more rapidly along the thickness direction than in length direction during stage – I. Faceted cubic shaped growth is facilitated on the surface of template which becomes more prominent and faster at higher temperature. Further, in presence of MnO_2_, liquid phase assisted grain growth takes place which also facilitates ionic diffusion increasing the kinetics of growth.^[1,2]^ At 1200^o^C the templates completely react with the matrix and dissolve.


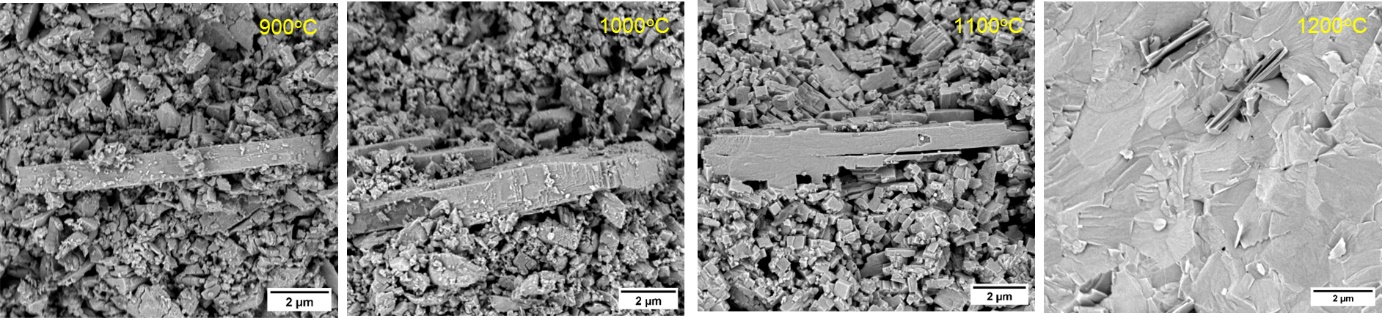


Figure S4. Microstructure development scheme showing nucleation and growth at the template surface for different temperatures without MnO_2_ addition (900^o^C, 1000^o^C, 1100^o^C and 1200^o^C dwelled for 15 minutes, all scaled to 2µm).


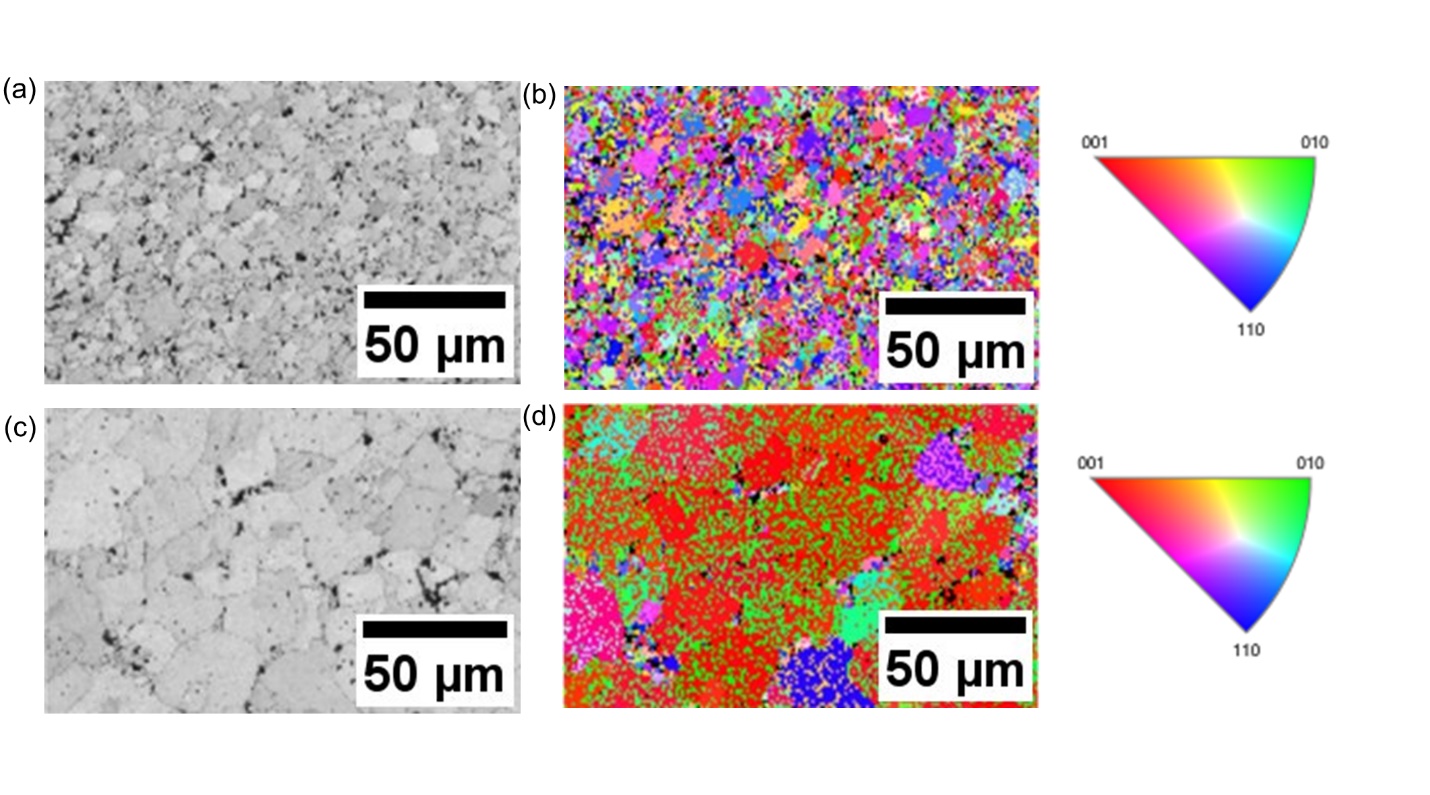


Figure S5. Band contrast image of (a) 0.1 MnO_2_ random and (c) 0.1 MnO_2_ textured ceramics with its respective EBSD at (c) and (d) along ‘*c*’ direction

1. **Calculation of Electrochemical Coupling (*K_31_* and *K_p_*)**

Electromechanical coupling is an important aspect to be considered for energy harvesters. Theoretically, K^2^ (Electromechanical coupling) is considered as (stored electrical or mechanical energy stored / (electrical or mechanical energy applied).^[3]^

Mathematically^[4,5]^ ,

*K_31_* = $\sqrt{\frac{A}{\left( A+1 \right)}}$ ; A = $\frac{\Pi}{2}\cdot\frac{fr}{f_{a}}\cdot\tan\left( \frac{\Pi}{2}\cdot\frac{\left( f_{r}-f_{a} \right)}{f_{a}} \right.)$ (1)

1/K_p_  = $\sqrt{0.398\cdot\frac{f_{r}}{f_{a}-f_{r}}+0.597}$ (2)

Table S2. Statistical analysis of *K_31_* from different samples of textured ceramics

| T(^o^C) | S1 | S2 | S3 | S4 | S5 | S6 | S7 | S8 | Mean | S.D | Confidence (95%) | UCL | LCL |
| --- | --- | --- | --- | --- | --- | --- | --- | --- | --- | --- | --- | --- | --- |
| 22 | 0.38 | 0.51 | 0.39 | 0.35 | 0.34 | 0.42 | 0.42 | 0.36 | 0.40 | 0.06 | 0.05 | 0.45 | 0.35 |
| 30 | 0.38 | 0.47 | 0.39 | 0.39 | 0.34 | 0.46 | 0.38 | 0.40 | 0.40 | 0.04 | 0.04 | 0.44 | 0.36 |
| 40 | 0.33 | 0.46 | 0.39 | 0.35 | 0.34 | 0.42 | 0.38 | 0.36 | 0.38 | 0.05 | 0.04 | 0.42 | 0.34 |
| 50 | 0.33 | 0.47 | 0.38 | 0.35 | 0.34 | 0.36 | 0.38 | 0.35 | 0.37 | 0.04 | 0.04 | 0.41 | 0.33 |
| 60 | 0.33 | 0.44 | 0.34 | 0.30 | 0.33 | 0.36 | 0.33 | 0.35 | 0.35 | 0.04 | 0.03 | 0.38 | 0.32 |
| 70 | 0.32 | 0.45 | 0.34 | 0.34 | 0.33 | 0.35 | 0.37 | 0.35 | 0.36 | 0.04 | 0.03 | 0.39 | 0.33 |
| 80 | 0.32 | 0.44 | 0.34 | 0.34 | 0.37 | 0.40 | 0.37 | 0.39 | 0.37 | 0.04 | 0.03 | 0.40 | 0.34 |
| 90 | 0.32 | 0.47 | 0.34 | 0.38 | 0.33 | 0.40 | 0.36 | 0.35 | 0.37 | 0.05 | 0.04 | 0.41 | 0.33 |
| 100 | 0.37 | 0.46 | 0.37 | 0.34 | 0.33 | 0.40 | 0.40 | 0.38 | 0.38 | 0.04 | 0.03 | 0.41 | 0.35 |
| 110 | 0.32 | 0.44 | 0.33 | 0.37 | 0.36 | 0.40 | 0.36 | 0.35 | 0.37 | 0.04 | 0.03 | 0.40 | 0.34 |
| 120 | 0.32 | 0.41 | 0.37 | 0.34 | 0.36 | 0.40 | 0.39 | 0.38 | 0.37 | 0.03 | 0.03 | 0.40 | 0.34 |
| 130 | 0.32 | 0.40 | 0.33 | 0.37 | 0.33 | 0.40 | 0.36 | 0.34 | 0.36 | 0.03 | 0.03 | 0.39 | 0.33 |
| 140 | 0.32 | 0.33 | 0.37 | 0.33 | 0.36 | 0.40 | 0.39 | 0.38 | 0.36 | 0.03 | 0.02 | 0.38 | 0.34 |
| 150 | 0.26 | 0.31 | 0.33 | 0.33 | 0.36 | 0.39 | 0.36 | 0.34 | 0.34 | 0.04 | 0.03 | 0.37 | 0.31 |
| 160 | 0.31 | 0.29 | 0.33 | 0.37 | 0.36 | 0.39 | 0.36 | 0.34 | 0.34 | 0.03 | 0.03 | 0.37 | 0.31 |
| 170 | 0.31 | 0.26 | 0.37 | 0.33 | 0.32 | 0.39 | 0.36 | 0.34 | 0.34 | 0.04 | 0.03 | 0.37 | 0.31 |
| 180 | 0.31 | 0.24 | 0.37 | 0.33 | 0.32 | 0.39 | 0.36 | 0.34 | 0.33 | 0.04 | 0.04 | 0.37 | 0.29 |
| 190 | 0.31 | 0.23 | 0.33 | 0.33 | 0.32 | 0.34 | 0.36 | 0.34 | 0.32 | 0.04 | 0.03 | 0.35 | 0.29 |
| 200 | 0.31 | 0.23 | 0.33 | 0.29 | 0.32 | 0.34 | 0.36 | 0.34 | 0.32 | 0.04 | 0.03 | 0.35 | 0.29 |
| 210 | 0.31 | 0.23 | 0.33 | 0.29 | 0.33 | 0.35 | 0.36 | 0.30 | 0.31 | 0.04 | 0.03 | 0.34 | 0.28 |
| 220 | 0.26 | 0.20 | 0.29 | 0.29 | 0.28 | 0.35 | 0.33 | 0.30 | 0.29 | 0.04 | 0.04 | 0.33 | 0.25 |
| 230 | 0.26 | 0.21 | 0.29 | 0.24 | 0.23 | 0.00 | 0.29 | 0.25 | 0.22 | 0.09 | 0.08 | 0.30 | 0.14 |
| 240 | 0.19 | 0.18 | 0.00 | 0.17 | 0.24 | 0.00 | 0.00 | 0.00 | 0.10 | 0.11 | 0.09 | 0.2 | 0.02 |

**Sample – S; Temperature- T; S.D – Standard Deviation; UCL – Upper confidence limit; LCL – Lower confidence limit;**

Table S3. Literature of KNN based Cantilever Structure Energy Harvesters

| Composition | V_PP_ OC  (V) | Power(_PP_)  (µW) | Frequency (HZ) | Power density  (µW/mm^3^) | Ref |
| --- | --- | --- | --- | --- | --- |
| 0.958(K_0.52_Na_0.48_)(Nb_0.96_Sb_0.04_)O_3_-0.036(Bi_0.5_Na_0.5_)ZrO_3_-0.006CaZrO3 | 12 | 48 | 80 | 0.96 | ^[6]^ |
| 0.1 MnO_2_ - (K_0.5_Na_0.5_)NbO_3_ | 7 | 16 | 90 | 0.32 | ^[7]^ |
| Na_0.52_K_0.435_Li_0.045_Nb_0.905_Ta_0.05_Sb_0.045_O_3_ | ~7.5 | 11.5 | 87.7 | 0.26 | ^[8]^ |
| 0.1 MnO_2_ KNN-BSZ | 14.2 | 50.5 | 130 | ~2 | This work |

Table S4. Comparison of real time application of cantilever type piezoelectric energy harvesters (PEH)

| Material System | Application | V_O/P_ (V) | P_O/P_ | P.D | Frequency (Hz) | Ref |
| --- | --- | --- | --- | --- | --- | --- |
| PZT -8 (MLCC) | Railway track Vibration | 9.14 V | 3.73 mW | 211 µW/g | 6 | ^[9]^ |
| ZnO - PVDF | Insole Pedometer | 14.6 | 85.2 µW | 21.32 µW/cm^2^ | 4 | ^[10]^ |
| PVDF/PVDF-TrFE | TPMS PEH |  | 6.23/39.75 µW |  | Self-Tuning  (Tire speed) | ^[11]^ |
| Textured KNN | Elephant type PEH | 14.2 | 50.5 µW | 2 µW/mm^3^ 500 µW/g | 130 | This Work |

1. Ceramic Synthesis


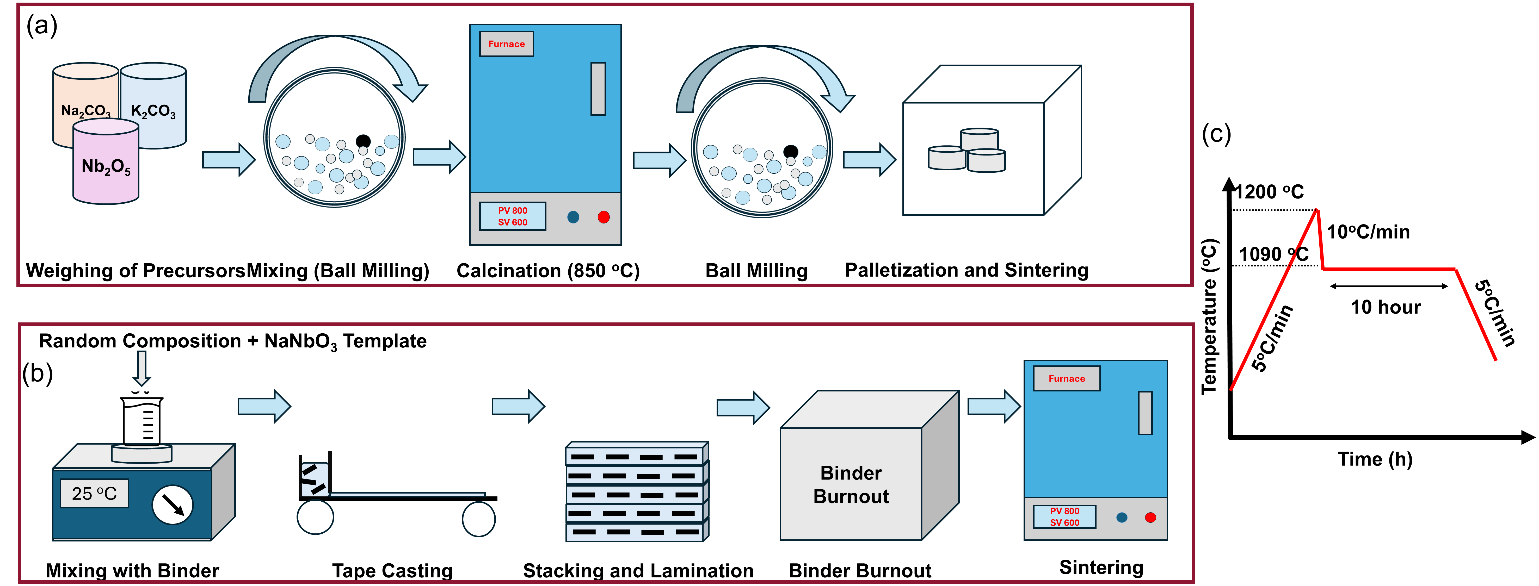


Figure S6. (a) Synthesis of Random composition. (b) Synthesis of Textured Composition and (c) Temperature profile used for multistep sintering

1. Design of Energy Harvester and Vibration velocity Measurement


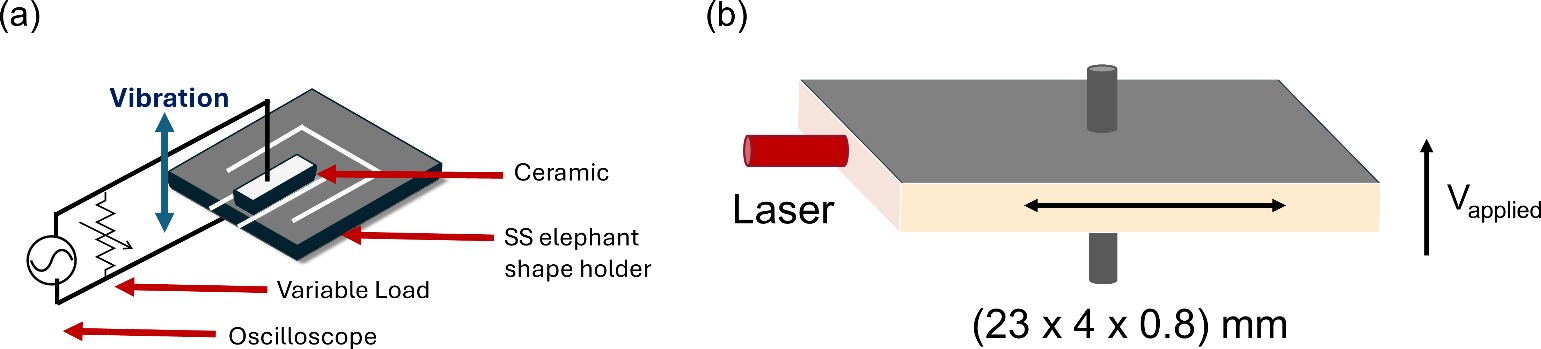


Figure S7. (a) Design of elephant shaped transducer for energy harvesting. (b) [31] mode ceramic for vibration velocity measurement.

References:

[1] H. Takao, Y. Saito, Y. Aoki, K. Horibuchi, *J. Am. Ceram. Soc.* **2006**, *89*, 1951.

[2] Y. Chang, S. F. Poterala, Z. Yang, S. Trolier-McKinstry, G. L. Messinge, *J. Mater. Res.* **2010**, *25*, 687.

[3] K. Uchino, *Adv. Piezoelectric Mater. Sci. Technol.* **2010**, 1.

[4] *IEEE Trans. Sonics Ultrason.* **1984**, *31*, 8.

[5] Y. Cheng, J. Xing, C. Wu, T. Wang, L. Xie, Y. Liu, X. Xu, K. Wang, D. Xiao, J. Zhu, *J. Alloys Compd.* **2020**, *815*, 152252.

[6] Y. Huan, X. Wang, W. Yang, L. Hou, M. Zheng, T. Wei, X. Wang, *J. Adv. Ceram.* **2022**, *11*, 935.

[7] M. Zheng, Y. Hou, X. Yan, L. Zhang, M. Zhu, *J. Mater. Chem. C* **2017**, *5*, 7862.

[8] M. Zheng, Y. Hou, L. Chao, M. Zhu, *J. Mater. Sci. Mater. Electron.* **2018**, *29*, 9582.

[9] Z. Min, C. Hou, G. Sui, X. Shan, T. Xie, *Micromachines 2023, Vol. 14, Page 892* **2023**, *14*, 892.

[10] A. Mahapatra, R. S. Ajimsha, M. O. Ittoop, A. Sharma, S. Karmakar, A. Shaikh, P. R. Sankar, P. Misra, *J. Alloys Compd.* **2023**, *960*, 170898.

[11] M. Momen, S. Ebrahimi-Nejad, M. Mollajafari, *J. Clean. Prod.* **2025**, *500*, 145255.
